# Supplementary material for: A UMLS-based spell checker for natural language processing in vaccine safety
Source: BMC Med Inform Decis Mak. 2007 Feb 12;7:3. doi: 10.1186/1472-6947-7-3 (PMC1805499; doi:10.1186/1472-6947-7-3)
Supplement: Additional file 13 — RAP application directory. Sets up directory for RAP (RDF application for PHP) source code [file 1472-6947-7-3-S13.gz › rap/doc/phpdoc/__filesource/fsource_resModel__resModelResSeq.php.html]

xml version="1.0" encoding="iso-8859-1"?


File Source for ResSeq.php


# Source for file ResSeq.php

Documentation is available at ResSeq.php

```
1. <?PHP
2. // ----------------------------------------------------------------------------------
3. // Class: ResSeq
4. // ----------------------------------------------------------------------------------


8. /**
9. * This interface defines methods for accessing RDF Sequence resources.
10. * These methods operate on the RDF statements contained in a model.
11. *
12. * <BR><BR>History:<UL>
13. * <LI>10-01-2004                : First version of this class.</LI>
14. *
15. * @version  V0.9.1
16. * @author Daniel Westphal <mail at d-westphal dot de>
17. *
18. * @package     resModel
19. * @access    public
20. ***/
21. class ResSeq extends ResContainer
22. {

24. /**
25. * Constructor
26. * You can supply a URI
27. *
28. * @param string $uri
29. * @access    public
30. */
31. function ResSeq($uri = null)
32. {
33. parent::ResContainer($uri);
34. $this->containerType=new ResResource(RDF_NAMESPACE_URI.RDF_SEQ);
35. }

37. /**
38. * Insert a new member into the sequence at the specified position.
39. * The existing member at that position, and all others with higher indexes,
40. * have their index increased by one.
41. *
42. * @param     integer    $index
43. * @param    object ResResource/ResLiteral    $resResource
44. * @return    boolean
45. * @access    public
46. */
47. function addAtIndex($index, $object)
48. {
49. //get a members index
50. $memberIndex= $this->getMembers();

52. //type this container, if it isn't already typed
53. if(!$this->hasProperty(new ResResource(RDF_NAMESPACE_URI.RDF_TYPE)))
54. $this->addProperty(new ResResource(RDF_NAMESPACE_URI.RDF_TYPE),$this->containerType);

56. //renumber all higher members
57. for ($i = count($memberIndex);$i >= $index ; $i--)
58. {
59. $this->removeAll($this->_getMembershipPropertyWithIndex($i));
60. $this->addProperty($this->_getMembershipPropertyWithIndex($i+1),$memberIndex[$i]);
61. }
62. //remove the old value at this position
63. $this->removeAll($this->_getMembershipPropertyWithIndex($index));
64. //add the new value
65. $this->addProperty($this->_getMembershipPropertyWithIndex($index),$object);

67. return $this;
68. }

70. /**
71. * Get the member at a given index
72. *
73. * @param     integer    $index
74. * @return    object ResResource/ResLiteral
75. * @access    public
76. */
77. function getMember($index)
78. {
79. $result=$this->listProperties($this->_getMembershipPropertyWithIndex($index));
80. if (isset($result[0]))
81. {
82. return $result[0];
83. }
84. else
85. {
86. return null;
87. }
88. }

90. /**
91. * Return the index of a given member of the sequence.
92. * If the same value appears more than once in the sequence, it is undefined
93. * which of the indexes will be returned.
94. * If the member is not found in this sequence, a value of 0 is returned.
95. *
96. * @param     object ResResource/ResLiteral $object
97. * @return    integer
98. * @access    public
99. */
100. function indexOf($object)
101. {
102. //check all members, until $object is found
103. foreach ($this->listProperties() as $statement)
104. {
105. $predicateLabel=$statement->getLabelPredicate();
106. if ($this->_predicateLabelMatchesMembershipProperty($predicateLabel))
107. {
108. if($object->equals($statement->getObject()))
109. //analyze the container membership property and return the index
110. return $this->_getMemberIndexNrFromMembershipPropertyLabel($predicateLabel);
111. }
112. }
113. //return 0 if $object wasn't found
114. return 0;
115. }

117. /**
118. * Remove the member at the specified index.
119. * All other members with a higher index will have their index reduced by one.
120. *
121. * @param     integer    $index
122. * @access    public
123. */
124. function removeAtIndex($index)
125. {
126. $memberIndex= $this->getMembers();


129. $this->removeAll($this->_getMembershipPropertyWithIndex($index));

131. for ($i = $index;$i < count($memberIndex); $i++)
132. {
133. $this->removeAll($this->_getMembershipPropertyWithIndex($i+1));
134. $this->addProperty($this->_getMembershipPropertyWithIndex($i),$memberIndex[$i+1]);
135. }
136. return $this;
137. }

139. /**
140. * Set the value at a given index in the sequence.
141. *
142. * If the index is not in the range of the sequence, false is returned
143. *
144. * @param     integer    $index
145. * @return    boolean
146. * @access    public
147. */
148. function set($index, $object)
149. {
150. if (!$this->hasProperty($this->_getMembershipPropertyWithIndex($index)))
151. return false;

153. $this->removeAll($this->_getMembershipPropertyWithIndex($index));
154. $this->addProperty($this->_getMembershipPropertyWithIndex($index),$object);
155. return true;
156. }
157. }
158. ?>
```

Documentation generated on Thu, 7 Jul 2005 13:43:52 +0200 by phpDocumentor 1.3.0RC3
